# Transforming Care: Exploring Consumer and Pharmacist Perceptions of Expanded Pharmacy Practice in Rural and Remote Communities

**DOI:** 10.3390/pharmacy13030071

**Published:** 2025-05-20

**Authors:** Selina Taylor, Shelby Joyce, Ruby Schembri, Josh Swain, Rachael Turiano, Beverley D. Glass

**Affiliations:** 1 Murtupuni Centre for Rural and Remote Health, James Cook University, Mount Isa, QLD 4825, Australia; 2 Pharmacy Department, James Cook University, Townsville, QLD 4811, Australia

**Keywords:** rural pharmacy, scope of practice, community pharmacy, access to healthcare, qualitative interviews

## Abstract

Accessing essential healthcare services presents a challenge for consumers living in rural and remote communities, leading to higher rates of chronic disease and poorer health outcomes. Community pharmacists are well positioned to address this lack of access; thus, this study aimed to explore the perceptions of rural and remote consumers and pharmacists with respect to community pharmacists expanding their services in these communities. Qualitative, semi-structured interviews were undertaken with consumers and pharmacists recruited from community pharmacies in the far north, north west, and central west Queensland. The Consolidated Framework for Implementation Research guided question development, with the responses deductively coded and thematically analysed. Thirteen pharmacists and twenty-three consumers were interviewed, with both groups citing the benefit of reduced wait times. Key barriers were pharmacist workload, time constraints, inadequate infrastructure, and limited consumer awareness of services. Pharmacists highlighted the need for better reimbursement models and professional collaboration, while consumers valued accessibility and convenience but were concerned about the costs of services. This study has highlighted the benefits of expanded pharmacy services as perceived by the key stakeholders: consumers and pharmacists. However, future research investigating a larger sample in more rural and remote locations is needed to ensure the successful implementation of sustainable funding models to deliver better access and health outcomes for consumers in these communities.

## 1. Introduction

Timely access to healthcare professionals is becoming increasingly challenging, particularly for rural and remote communities [1]. Consumers residing in these areas reported waiting longer than they felt acceptable for healthcare in comparison to their metropolitan counterparts [1]. This delay in healthcare access has led to higher rates of chronic diseases and poorer health outcomes compared to those in urban areas [2]. Unique challenges, such as geographic isolation, limited infrastructure, low population density, workforce shortages, and increased healthcare costs, have exacerbated the delays, resulting in increasing rates of hospitalisation [2]. An investigation into public hospitals revealed a sustained growth in emergency department presentations, with hospitals currently operating at full capacity, increasing the risk of an unsustainable healthcare system [3]. Australian studies have shown that the wellbeing of the GP workforce has also been declining, with 7 in 10 GPs experiencing feelings of burnout in 2023 due to overwhelming demands, especially in rural towns [4], leaving many communities at risk of losing access to essential local healthcare services.

Community pharmacists are often the first point of call regarding health advice for patients in rural areas; however, practising to their full scope is often neither utilised nor understood [5]. Rural community pharmacists have a unique opportunity to expand the scope of their practice to address current gaps in rural healthcare [6]. In Queensland, Australia, the role of the pharmacist has evolved significantly, with the Extended Practice Authority allowing pharmacists to administer immunisations and prescribe antibiotics for uncomplicated urinary tract infections (UTI) following specialised training [7]. This evolution has led to the incorporation of these service delivery models into everyday practice, highlighting pharmacists’ expertise, capabilities, and commitment to expanding their role further within community pharmacy.

Further developments in expanded practice are apparent internationally, with consumers reporting positive experiences with community pharmacy-based expanded services. In the United Kingdom (UK), a study conducted by Lowrie et al. found that patients identified community pharmacies as an appropriate setting for a heart failure management service. This service was reported to positively impact their care, with pharmacists enhancing their own understanding of the condition, while also effectively addressing a gap in supporting patients self-management [8]. A survey by Navarrete et al. conducted in Japan, Thailand, and Canada found that pharmacists who provided a sexual reproductive health (SRH) service were interested in expanding their roles; however, most pharmacists expressed the need for additional training in SRH services (Japan = 79%; Thailand = 91%; Canada = 84%) [9]. In Australia, a study conducted in rural Queensland by Taylor et al. stated that 98% of consumers (*n* = 55) would prefer to go to the pharmacy for their ear conditions rather than to a GP [10]. This is attributed to the fact that 87% of consumers valued the in-depth information provided by the pharmacist, which enhanced their understanding of their treatment [10]. Beyene et al. found that community pharmacists were confident in their ability to provide a community pharmacy anticoagulation management service, which increased their job-satisfaction and enhanced pharmacist–patient relationships, allowing them to assist patients with other aspects of their health [11]. These studies have demonstrated that pharmacists have the capability to positively impact patient outcomes by expanding their scope of practice, [12] alleviating pressure on emergency departments reducing patient wait times and the overall strain on the healthcare system [13].

Nevertheless, in Australia, despite the expertise and well-recognised role of rural community pharmacists, there is a lack of access to structured or funded care models to support the expansion of their practice, restricting efforts to reduce health disparities in these regions [5]. The current literature highlights a notable gap between the trial phase of a service and its successful implementation in rural communities. Expanded practice trials such as LISTEN UP (Locally Integrated Screening and Testing Ear and Aural Programme) and a Chronic Obstructive Pulmonary Disease (COPD) case finding, although resulting in favourable outcomes, have not been integrated into routine pharmacy practice [10,14]. This disconnect may be due to insufficient knowledge surrounding funding allocation and inadequate structural frameworks for pharmacists to implement these services [10]. Additionally, these studies have often failed to include the perspectives of both consumers and pharmacists, both of whom are key stakeholders in the effective delivery of expanded services in these rural communities. Therefore, this study aimed to explore rural and remote consumer and pharmacist perceptions of community pharmacists expanding their services in rural communities in Queensland, specifically in Barcaldine, Emerald, Mount Isa, and Weipa.

## 2. Materials and Methods

### 2.1. Study Design

An ethnographic lens of rural culture was applied to this descriptive qualitative study. This involved the researchers immersing themselves in the remote communities in which they were collecting data. Consumers and pharmacists were interviewed using an in-depth semi-structured interview process.

### 2.2. Participants, Setting, and Recruitment

During January–February 2023, rural consumers and pharmacists at community pharmacies in Barcaldine, Emerald, Mount Isa, and Weipa, Queensland were conveniently sampled to participate in an interview by face-to-face invitation. Convenience sampling allowed for collection of data from a broad range of consumers who visit pharmacies, rather than those purposely selected who may have more interest in pharmacy services. Two pharmacists and fourteen consumers were invited but declined to participate due to time constraints. These communities were selected for the study to allow for a diverse data spread across remote Queensland. To be eligible for inclusion in the study the participants invited were required to be 18 years or older, understand and speak English and be living in the remote community in which the research was conducted.

### 2.3. Procedure and Semi-Structured Interview

All eligible participants that agreed to participate were given a study information leaflet detailing the purpose of the study, a confidentiality statement and consent form. Participants were interviewed face-to-face in the community pharmacies, with the interviews audio recorded via voice recorder and the data de-identified in the transcription process. Demographic data, including gender, location, time living in the rural location, and years of experience (pharmacists only), were also collected.

The schedule of interview questions was developed prior using the Consolidated Framework for Implementation Research (CFIR) to ensure that the data aligned with the aim of the study (Appendix A) [15]. The CFIR is a collection of constructs consisting of individual subconstructs that correlate with successful implementation of innovations across a range of applications [15]. Tailoring questions based on the CFIR produced quality data that either supported or hindered the implementation of expanded services. This allowed for recommendations to be formulated that were specific to the facilitation of expanded services in rural community pharmacies [15]. The interview schedule was piloted with three experts in qualitative methodology to ensure content validity. Table 1 summarises the domains or constructs of the CFIR specific to implementation of expanded pharmacy services in rural communities [15].

### 2.4. Data Analysis

**Figure 1:**
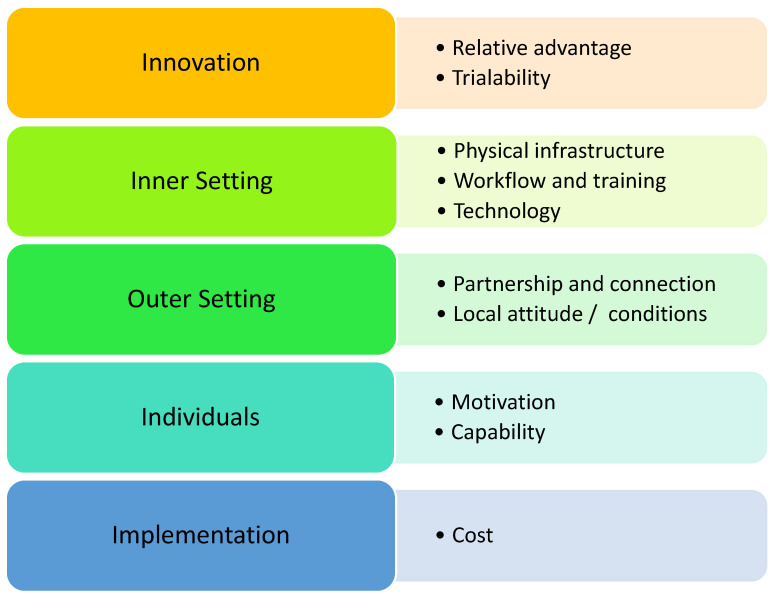
Identified themes mapped to the CFIR framework.

The qualitative data were analysed thematically utilising the Braun and Clarke thematic analysis framework, which includes familiarisation with the data, the generation of initial codes, searching for themes, reviewing themes, defining and naming these themes, and writing up the findings [16]. The raw, de-identified transcripts were transcribed verbatim to remove any errors from the automated software transcription, which allowed the researchers to familiarise themselves with the data. The transcripts were then coded deductively against the CFIR framework [15], allowing for the emergence of themes throughout the data. The themes generated reflected the core CFIR domains, namely, innovation, inner setting, outer setting, characteristics, and implementation, and the generated codes in the data were grouped under their respective CFIR construct [Figure 1] [15]. The themes were reviewed to verify that the codes categorised under each theme were relevant to the CFIR constructs. This approach refined patterns from the qualitative data into pre-existing, evidence-based themes, allowing for the extrapolation of findings to reach conclusions relevant to the aim of the study. The data gathered were integrated under the constructs of the CFIR, comparing the perspectives of the consumers and pharmacists using the “following the thread” protocol [17]. This method of data amalgamation is well established and has been identified as a validated framework for the integration of various datasets [17]. Objectivity, assumed knowledge, and bias were minimised, and four participants were engaged in a member checking process to ensure that their code/theme interpretations were an accurate representation of their voice.

After 28 interviews, data saturation was achieved, and no new themes had emerged for four interviews; however, the remaining eight participants who had volunteered to participate were included in the interview and analysis process to ensure that no emergence of new linkages or themes occurred. The research team met regularly throughout the data collection phase to engage in critical self-reflection, promote continuous self-awareness, and reflect upon their biases and values to ensure reflexivity.

### 2.5. Ethics Approval

This study has been approved by the Human Research Ethics Committee, James Cook University (reference number: H8969).

## 3. Results

A total of 36 participants, consisting of 13 pharmacists and 23 consumers, were interviewed face-to-face. Interviews varied in length from 15 to 45 min, with an average duration of 25 min. Table 2 presents the characteristics of both participant groups. The results, mapped against the CFIR domains, are presented, with quotations cited as PX (pharmacist) or (CX) (consumer).

### 3.1. Innovation

The innovation domain evaluates the feasibility, relevance, and effectiveness of new services, focusing on potential success and sustainability.

#### 3.1.1. Relative Advantage

Both pharmacists and consumers emphasised the importance of expanding pharmacy services, especially in rural areas, where healthcare access is limited. The perceived advantages of expanded services over traditional healthcare options were primarily convenience and reduced wait times.

“*It takes like 6 weeks to get into the doctor. Pharmacists are the most accessible and consistent healthcare here*.”

(C4-MMM4)

“*Until you actually live here, you don’t understand…going to the doctor is not an option, it’s not even on the table because you can’t get in.*”

(C2-MMM7)

Many participants turned to online doctors as a last resort when timely GP appointments were unavailable.

“*Expanded practice is definitely the way forward in communities where the wait lines are long and then they have to turn to online doctors who often prescribe the wrong medicines.*”

(P3-MMM4)

When online services failed to meet expectations or provide timely solutions, many consumers mentioned having no other option but to resort to emergency departments.

“*I know a lot of people show up at the emergency department with very GP-appropriate problems*.”

(C3-MMM4)

Pharmacy services were repeatedly mentioned to be beneficial for non-emergency needs, such as repeat prescriptions, as consumers expressed frustration over needing a GP appointment for long-term medications.

“*Even if they could do repeats on scripts…the pharmacist would be able to give you a prescription. Surely that is something they can do?*”

(C5-MMM4)

#### 3.1.2. Trialability

Pharmacists indicated that trialing new services allowed them to assess effectiveness and gauge consumer acceptance on a smaller scale. Although the uptake of new services was expected to be slow at first, pharmacists described existing services having a moderate–high uptake once the services became more familiar to the community.

“*The idea of getting a pharmacist to vaccinate you was confronting at the time, but as it became normalised, we do the majority of vaccinations now, and the overall vaccinated population has increased with that access*.”

(P1-MMM7)

Beyond vaccinations, pharmacists also commented on the success of other pharmacy services.

“*We do UTI consultations, which are very popular.*”

(P7-MMM7)

“*This morning, I did a B12 injection, and the customer was just so happy that I could do it, because you’re waiting about 6 weeks to see a doctor at the moment*.”

(P4-MMM4)

### 3.2. Inner Setting

The inner setting domain examines internal factors within pharmacies that influence the adoption and implementation of new services. These factors are crucial to determining how effectively new services can be integrated into the daily operations of a pharmacy.

#### 3.2.1. Physical Infrastructure

Both consumers and pharmacists acknowledged the limitations in physical space within pharmacies to accommodate new services, emphasising the need for significant investments in infrastructure.

“*I didn’t design the business 13 years ago to have space or adequate rooms for this*.”

(P13-MMM7)

“*Before even enrolling into the prescribing course, you’re looking at the consult room going, where would I even do this, this isn’t sufficient enough*.”

(P2-MMM7)

Unexpected concerns were also raised among consumers concerning compromising existing stock to accommodate this layout as rural pharmacies often serve as the only source of specific health products.

“*It would be hard to create the space for it, you don’t want to take away products, we don’t have much access to many products here*.”

(C1-MMM7)

#### 3.2.2. Workflow and Training

Pharmacists reported concerns related to time management, staffing, and administrative burdens that complicate implementation. Their current workload time constraints were also a recurring issue, with pharmacists describing themselves as being time-poor, leaving them little time to take on additional tasks. This highlighted the challenge of balancing patient care with operational demands.

“*We are so time-poor. Last week, a man wanted to consult with me, but I was too busy, and he left. There’s no way you can do a 10-15-min consultation with only one pharmacist on, and you cannot leave a dispensary unsupervised for that long*.”

(P3-MMM4)

“*We’re already stretched thin, so adding more services without more staff or resources is going to be tough*.”

(P2-MMM7)

Both consumers and pharmacists highlighted the lack of adequate staffing as a major barrier to implementing additional services.

“*Occasionally, the pharmacy has had to shut the doors because the pharmacist is away or sick*.”

(C2-MMM7)

“*With more pharmacists, we wouldn’t feel rushed and could spend more time with each patient.*”

(P2-MMM7)

The need for additional training also posed challenges, particularly in rural areas where travel for training is often required.

“*I would have to get a locum to come if I had to go to Brisbane for training.*”

(P8-MMM7)

#### 3.2.3. Technology

Pharmacists expressed concern about whether current software systems could handle the expansion, particularly regarding access to pathology results and complex data management. This concern was shared by consumers who were uncertain about how technology could support expanded services.

“*We need to ensure that the software can support it. We need a system for booking appointments, recording interactions, communicating with doctors for tests-that doesn’t exist yet*.”

(P5- MMM7)

“*Yeah I’m not sure how they will make sure they have what the doctors have.*”

(C14-MMM4)

### 3.3. Outer Setting

The outer setting domain explores the external factors influencing the adoption of expanded pharmacy services, highlighting the broader context in which community pharmacies operate.

#### 3.3.1. Partnership and Connection

Interprofessional relationships emerged as a significant theme. Both pharmacists and consumers discussed the importance of these relationships in shaping the acceptance of expanded services. While some felt confident that local GPs would welcome additional support, others anticipated resistance and were unsure as to whether these changes might lead to more challenges than benefits.

“*Doctors think that pharmacists are trying to cut their grass*.”

(C19-MMM7)

“*I don’t see why it should impact our dynamic, we’re not taking away their entire patient base. With UTI consults, I’ve heard from doctors here saying how helpful it has been*.”

(P2-MMM7)

“*I think doctors also don’t want to give up some of their role. Some of our doctors have that mentality of, I’m the doctor, you are just a pharmacist… there would have to be a big conversation in the medical centre before making changes because you’ve got to rely on them to send eScripts, you don’t want them to stop all that and lose that service at all.*”

(C1-MMM7)

Pharmacists voiced concerns about potential pushback, especially in small towns where maintaining strong professional relationships is critical.

“*I do have this fear of pushback from other healthcare professionals, I feel like it will be detrimental, especially in a small town.*”

(P5-MMM7)

This created a sense of indecisiveness, as the desire to expand pharmacy services was tempered by the need to carefully navigate existing professional dynamics.

#### 3.3.2. Local Attitude and Conditions

Consumers expressed high levels of confidence in pharmacists’ abilities and valued the personalised care they received, attributing this to the pharmacists’ consistent presence.

“*A pharmacist already knows our history. A lot of the bush people think, I’m just a number, but you have that personalisation here*.”

(C8-MMM7)

This stability was viewed as a major advantage, with ongoing relationships enhancing consumer confidence in adopting new services. Consumers reported feeling more comfortable with pharmacists due to these long-standing relationships.

In contrast, consumers frequently described their interactions with locum doctors as impersonal and inconsistent.

“*They have lots of locums and every time I go, there’s a new doctor, so you have to go through your whole medical history again, which feels very impersonal.*”

(C6-MMM4)

An unexpected finding was the lack of consumer awareness about the range of services available at pharmacies, which was identified as a significant barrier to the broader adoption of these services.

“*I’ve never heard of the UTI prescribing before-that would’ve saved me a kidney infection.*”

(C11-MMM7)

Pharmacists echoed this, emphasising the need for better promotion of available services to increase awareness.

“*People are just so unaware of what we can do and we, as pharmacists, aren’t very good at promoting it either*.”

(P3-MMM4)

### 3.4. Individiuals

The individuals domain focuses on the individuals driving the adoption of innovations, ensuring that they receive the support needed for success.

#### 3.4.1. Motivation

Pharmacists were motivated to expand services to serve the needs of their communities and were excited to contribute to improved patient outcomes. Many pharmacists viewed the integration of existing expanded pharmacy services as an encouraging step forward.

“*The UTI pilot has been a nice entry into the idea of expanded scope, and the fact that most of the community will be grateful for having us as an option is motivating.*”

(P2-MMM7)

However, some pharmacists struggled to adapt to these changes, expressing difficulty in keeping up with the growing scope of their responsibilities.

“*Coming into this degree, I wanted to be a pharmacist, I didn’t want to do medicine and it’s far too much, it’s hard to keep up*.”

(P6-MMM4)

#### 3.4.2. Capability

Pharmacists felt positively about offering expanded practice services but were conservative about the extent of services they could deliver, focusing on implementing services that incorporated existing roles such as consultations and screening services.

“*If we start offering too many services, patients will be seeing multiple professionals, I don’t believe that’s the best approach. While we have the capability to manage chronic care, I just don’t think it’s within our role as pharmacists to take on that responsibility.*”

(P1-MMM7)

In contrast, consumers suggested a wider range of services, including those beyond what pharmacists typically provide.

“*Anything they can do here at the pharmacy I think should be done*.”

(C21-MMM7)

Despite this, some pharmacists felt that their skills and knowledge were underutilised and that, with adequate resources and support, they have the capability to provide more services. Comparatively, consumers felt pharmacists were already well utilised, noting how pharmacists often undertake roles that are more than what is expected of their usual roles.

“*Pharmacists always go above and beyond; they’ve always been really helpful.*”

(C17-MMM7)

The capability of pharmacists to expand their scope was largely connected to their training and confidence in acquiring new skills. Pharmacists emphasised the need for hands-on training and expressed a preference for in-person learning over online alternatives typically offered in rural areas.

“*For myself there is a bit of hesitancy. I don’t think we are fully equipped at the moment. If they’re going to expand the scope, we need appropriate training*.”

(P4-MMM4)

Despite these concerns, many pharmacists felt equipped, citing instances where their interventions could have prevented more serious health issues.

“*Straight away I could tell it was shingles, but there wasn’t anything I could do. I had to send him to the hospital. He came back with a script for antivirals, the same ones I would have prescribed*.”

(P3-MMM4)

### 3.5. Implementation

The implementation domain focuses on the practical measures required to put an innovation in place, addressing aspects such as planning, executing, and sustaining the new practice.

#### Cost

Pharmacists noted significant financial challenges associated with implementing expanded services, particularly in rural areas, where infrastructure upgrades were necessary.

“*We would be looking at around $150,000 in renovations and equipment*.”

(P13-MMM)

“*Even with the proposed grant they’re doing, it is not nearly enough*.”

(P11-MMM7)

Pharmacists suggested that financial assistance, such as grants and support from pharmacy organisations including The Pharmacy Guild of Australia, as well as government funding for expanded services would be required for the services to be sustainable in the future.

“*The Guild and government grants are necessary for expanded services. Otherwise, we just can’t afford to run these programs*.”

(P1-MMM7)

Additionally, a generational divide emerged in consumers’ willingness to pay for services, with younger generations generally more open to paying, while older individuals tended to be more reluctant.

“*I think the younger generation will pay, the older generation won’t. They’re still in that mentality of money is really hard to come by. Whereas the younger generation thinks, you pay for what you want*.”

(C10-MMM7)

Most consumers preferred fixed service charges over time-based fees, with the majority willing to pay between AUD 20–AUD 50 for each service, as long as it remained cheaper than visiting a doctor.

“*To see a GP you’re looking at around AUD $89 so anything below that I would pay for.*”

(C4-MMM4)

“*It’s cheaper and quicker to see the pharmacist than waiting weeks and paying more for a doctor’s visit.*”

(C7-MMM7)

Pharmacists shared concerns about the feasibility of covering the costs associated with offering expanded services, both for the pharmacy and for patients. They emphasised how even small increases in service fees have, in the past, significantly influenced patient decisions.

“*A patient came in on Sunday for a vaccination and we told him the price was higher, so he decided to come back on Monday*.”

(P9-MMM7)

“*Since starting to charge for monitor-related services we’ve noticed a decrease in people utilising this service.*”

(P12-MMM7)

## 4. Discussion

This study investigated consumers’ and pharmacists’ perceptions across diverse rural locations in Queensland to gain insight into the views of these populations on expanding pharmacy services. Consumers and pharmacists recognised that there is a deficit in access to healthcare professionals in rural locations and believed that pharmacists have the potential to reduce this gap. This study highlights consumers’ perception of pharmacists as knowledgeable, accessible, and trustworthy health professionals. Additionally, consumers would be willing to utilise expanded practice services if offered by their pharmacy. However, consumers still viewed doctor and pharmacist roles as distinct, preferring to seek only specific services such as repeat prescriptions, short course antimicrobials, and chronic disease screening from their pharmacist.

On a global scale, community pharmacists are providing various expanded practice services; however, in Australia, such services are limited [6]. This study demonstrated consumer support for expanded pharmacy services in rural and remote areas. The types of services, frequently suggested by consumers, focused on chronic disease management for conditions such as diabetes mellitus, respiratory issues (particularly asthma), cardiovascular disease, and dermatological conditions. These findings are consistent with previous Australian studies, which showed a high demand for pharmacists’ involvement in chronic disease management [6]. Given that over 60% of Australians are expected to develop a chronic disease at some stage of their life, these recommendations align with the current health needs expressed by consumers [18]. Additionally, our results reflect that services which predominantly support the needs of the older population were more commonly proposed among consumers, whereas conditions that typically affect younger demographics, such as sexually transmitted infections and drug and alcohol support services, were seldomly recommended for implementation [6]. However, it was found that consumers often lacked awareness regarding the range of services that pharmacies offered, as well as the pharmacist’s scope of practice. Limited awareness and consequent underutilisation of pharmacy services is an issue, which is not exclusive to Australia. A systematic review of expanded pharmacy services conducted in the UK also noted that lack of public awareness was an influential factor for inadequate consumer participation in pharmacy services [19]. Furthermore, this review supports our findings that consumers recognised the need for increased advertising and promotion of the services offered within the pharmacy to increase uptake [19].

Previous studies conducted in Malta have found that consumers do not fully recognise the value of a pharmacist and view them more as “glorified shopkeepers” [20]. However, the findings of this study contradict this, with all consumers indicating a positive relationship with their pharmacist. Consumers acknowledged that this relationship influenced their preference for receiving expanded practice services from the pharmacist rather than their GP. In addition to feeling valued, consumers noted that accessibility and convenience were key advantages of pharmacists offering expanded practice services. Studies in both New South Wales, Australia, and the UK resonate with these findings, proving that convenience and accessibility were key facilitators for consumer utilisation of expanded practice services [19,20,21]. Unsurprisingly, this is particularly significant given that over 50% of people living in rural areas work full time [22]. As work demands continue to grow and schedules overload, convenience becomes a crucial determining factor for consumers choosing where and when to access healthcare.

Most consumers indicated that they would be willing to pay for expanded pharmacy practice services, valuing the convenience and accessibility that these services provide. However, with rural and remote residents earning a weekly household income that is approximately 30% less than those who live in metropolitan locations, consumers expressed concerns that cost would be a significant barrier to accessing these services [23]. Overall, our results highlight that pricing comfort for consumers is multifaceted and largely influenced by the nature of the service, the level of pharmacist involvement, and the duration of the consultation. This observation is consistent with an Australian study exploring consumer perceptions of expanded practice in rural community pharmacy, suggesting that further research into the willingness to pay for specific expanded services may be valuable [6]. While all consumers agreed that they would be confident in utilising expanded practice services if offered by their pharmacy, the majority preferred to seek specific services that involved a short consultation with the pharmacist, as they saw doctor and pharmacist roles as distinct. These findings align with a study in Scotland that demonstrate that even those who see their pharmacist as a first point of call for medical related questions, still turn to their GP for more in-depth consultations [8,19]. Furthermore, the same Scottish study found that consumers will often use an initial consultation with the pharmacist to determine if they should self-refer to their GP [8,19].

The majority of pharmacists had a positive attitude towards expanded practice services and felt confident in their ability to provide them. However, many pharmacists expressed frustration towards current issues facing rural communities such as long wait times for healthcare and restrictive prescribing legislation, strongly advocating for change. This sentiment aligns with that of the Pharmaceutical Society of Australia, which asserts that “red tape and regulations get in the way of pharmacists and must go” [24]. Learnings from pharmacist prescribers in New Zealand could further support the development of expanded practice in rural Australia, with services including responding to medicine queries, medicine use review, medicine therapy assessments, and medicine optimisation being common tasks provided by pharmacists in prescribing roles [25]. Importantly, pharmacists reported that the key perceived benefits for patients included improved health outcomes and understanding of medicines, and benefits for the health services included supporting the workloads of busy primary healthcare staff [25].

Currently, many pharmacists feel underutilised in their roles, despite their qualifications and skills, and are motivated to up-skill to provide these expanded services. A Western Australian study investigating pharmacist vaccination services found that pharmacists reported significant professional satisfaction from undertaking these roles [26]. In addition to professional gratification, pharmacists felt that providing these services created opportunities to build therapeutic relationships and improve communication between healthcare professionals, ultimately benefiting consumers [26]. However, despite positive consumer demand, pharmacists acknowledged that workload and time constraints are major barriers to implementing expanded practice services. Other concerns that were frequently raised by pharmacists regarded adequate funding and reimbursement that reflects the effort and time involved for pharmacist consultations. Pharmacists emphasised the need for effective finance models as an incentive to provide expanded practice services to ensure sustainability [27].

The findings of this study have reinforced the well-documented issue that individuals who live in rural and remote locations experience poorer health outcomes and limited access to healthcare services compared to their metropolitan counterparts, highlighting the implications for future expanded practice and recommendations regarding targeted interventions to address these disparities.

### Strengths and Limitations

The strength of this study is that is presents the perspectives of the key stakeholders, namely, both the customers and the pharmacists, interviewed in person in their rural location, which allowed for an in-depth exploration of pharmacists expanding their practice. This study’s limitations include the study design, involving the recruitment of consumers within a pharmacy setting, which may have led to the inclusion of participants who regularly visit these pharmacies and have a more favourable view of the pharmacists, therefore potentially introducing an unintentional bias to the findings. In addition, although recruitment included four towns across the far north, central west, and northwest Queensland, these findings might not be generalisable to other rural communities, both nationally and internationally. Findings may also have limited generalisability in other countries, as pharmaceutical services are governed by specific regulations, and factors such as healthcare access, interprofessional relationships, health literacy, and the skills and knowledge of pharmacists may vary significantly across countries. Economic, cultural, and geographical differences could further limit the generalisability of these results to other contexts.

## 5. Conclusions

Limited access to healthcare professionals in rural and remote areas contribute to poorer health outcomes and higher rates of chronic disease for these populations [1,2]. This study has highlighted common perspectives held by both rural consumers and pharmacists on potentially expanding pharmacy services to address the healthcare gaps in these communities. Consumers, although often unaware of the expanded services offered, were positive about these services, while pharmacists, despite the challenges of workload and funding, were keen to provide these services to better serve the needs of their communities. Both agreed on community pharmacists’ unique position to offer expanded services due to their convenience of access. Insights into the barriers and facilitators of implementing expanded pharmacy services have been provided. Future research will be required to prioritise the development of effective funding and reimbursement models, as well as to increase consumer awareness through targeted education strategies. Ultimately, it has been determined that expanded practice services can play a crucial role in transforming healthcare delivery for Australians residing in rural and remote communities.

## Figures and Tables

**Table 1:** CFIR domain definitions applied to the rural setting [15].

Domain	Definition
Innovation	Service implemented within the rural community pharmacy
Inner Setting	Community pharmacy in which the expanded services will be implemented, examining the infrastructure, compatibility, and resources required for the successful implementation of the innovation
Outer Setting	Rural community in which the community pharmacy is located, including the individuals, policies, and relationships
Individuals	Roles and characteristics of individuals directly or indirectly associated with the expanded pharmacy services implementation
Implementation	Strategies used to aid in the successful implementation of expanded pharmacy services

**Table 2:** Participants characteristics—pharmacists (*n* = 13) and consumers (*n* = 23).

Category	Variable	Number
Gender	Female	21
	Male	15
Location	Barcaldine	6
	Emerald	15
	Mount Isa	8
	Weipa	7
Time spent living in a rural/remote location	0–1 years	4
	2–5 years	6
	6–10 years	8
	11–15 years	3
	15+ years	15
Years of experience as a pharmacist	0–1 years	4
	2–5 years	4
	6–10 years	1
	11–15 years	2

## Data Availability

No additional data are available due to privacy restrictions.

## Abbreviations

The following abbreviations are used in this manuscript:

CFIR	Consolidated Framework for Implementation Research
COPD	Chronic Obstructive Pulmonary Disease/Disorder
GP	General Practitioner (Doctor)
SRH	Sexual Reproductive Health
UK	United Kingdom

## Appendix A

### Appendix A.1. Interview Schedule for Consumers

Thank you for consenting to participate in this research project about expanded services through community pharmacies. This study aims to investigate rural and remote consumers’ points of view and insight regarding pharmacists expanding the scope of their practice to provide additional services in pharmacies to better the health of the community. Please be aware that taking part in these interviews is completely voluntary and that at any part throughout the interview or study, and without explanation or prejudice, you can choose to cease to participate. Please reaffirm your consent before we begin.

- How long have you lived in this or another rural/remote area?
- What do you think the most prevalent diseases or health conditions are in the community? Prompts diabetes, heart disease, injuries, mental health.
- Do you think your pharmacy could provide any additional services to address this health conditions, what sort of services? Prompts ○ What do you think will help improve some of the most prevalent conditions such as diabetes/asthma? ○ Education-inhaler technique, weight management, diabetes testing.
- Does your pharmacy currently provide any expanded services your community pharmacy provides and what are these services? Prompts Vaccinations, blood pressure checks, webster packing, diabetes testing.
- Do you think local people would utilise expanded services if the pharmacy was able to provide them? Why? What are the current options for healthcare in the community? Prompts ○ GPs, emergency departments, specialist services.
- What do you think might be barriers to pharmacies providing expanded services? Prompts ○ Financial constraints; ○ Staff availability ○ Time constraints; ○ Space to provide education/tests, etc.
- Do you feel confident about pharmacists delivering expanded services? Are there any factors/support that could be provided to you that would make you feel more confident receive these expanded services? HREC Amendment Form v1.0 01.2020 12 Prompts ○ More training ○ Better access to resources/education ○ Telehealth-access to specialists/doctors.
- Do you think people would be willing to pay for pharmacists to provide expanded services? What value? Prompts ○ Value per hour; ○ Which services would be more highly valued compared to others?

### Appendix A.2. Interview Schedule for Pharmacists

Thank you for consenting to participate in this research project about pharmacist expanded services through community pharmacies. This study aims to investigate rural and remote practising pharmacists’ points of view and insight regarding pharmacists expanding the scope of their practice to provide additional services in pharmacies to better the health of the community. Please be aware that taking part in these interviews is completely voluntary and that at any part throughout the interview or study, and without explanation or prejudice, you can choose to cease to participate. Please reaffirm your consent before we begin.

- How long have you been practising and how long have you been practising in rural/remote areas?
- What services would you like to offer that would benefit the community based on the most prevalent diseases in the community Prompts ○ What do you think will help improve some of the most prevalent conditions such as diabetes/asthma? ○ Education—inhaler technique, weight management, diabetes testing.
- How many patients (rough estimate on number) do you think use the expanded services your community pharmacy provides and what are these services? Prompts ○ Do you provide ear checks, BP monitoring?; ○ Diabetes testing?
- What resources are available to help you implement expanded services in your pharmacy? Prompts ○ CPD training—online, in person.
- What do you think are the real and perceived barriers when implementing these expanded services? Prompts ○ Financial constraints; staff availability ○ Time constraints; lack of training resources to improve skills/knowledge ○ Space to provide education/tests, etc.
- Do you feel confident delivering expanded services? Are there any factors/support that could be provided to you that would make you feel more confident to deliver these expanded services? Prompts ○ More training; better access to resources/education ○ Telehealth-access to specialists/doctors, etc.; more funding, staff, infrastructure.
